# Investigations of the Optical Properties of GaNAs Alloys by First-Principle

**DOI:** 10.1038/s41598-017-17504-w

**Published:** 2017-12-11

**Authors:** Damir Borovac, Chee-Keong Tan, Nelson Tansu

**Affiliations:** 1 Center for Photonics and Nanoelectronics, Department of Electrical and Computer Engineering, Lehigh University, Bethlehem, PA 18015 USA; 2 Department of Electrical and Computer Engineering, Clarkson University, Potsdam, NY 13699 USA

## Abstract

We present a Density Functional Theory (DFT) analysis of the optical properties of dilute-As GaN_1−x_As_x_ alloys with arsenic (As) content ranging from 0% up to 12.5%. The real and imaginary parts of the dielectric function are investigated, and the results are compared to experimental and theoretical values for GaN. The analysis extends to present the complex refractive index and the normal-incidence reflectivity. The refractive index difference between GaN and GaNAs alloys can be engineered to be up to ~0.35 in the visible regime by inserting relatively low amounts of As-content into the GaN system. Thus, the analysis elucidates on the birefringence of the dilute-As GaNAs alloys and comparison to other experimentally characterized III-nitride systems is drawn. Our findings indicate the potential of GaNAs alloys for III-nitride based waveguide and photonic circuit design applications.

## Introduction

III-nitride semiconductors have been extensively studied in the past decades and have been in the epicenter of semiconductor research in recent years^1–4^. The binary (Al, Ga, In)N alloys and their ternary and quaternary common-anion alloys possess remarkable optoelectronics, chemical, and tribological properties^5,6^ leading to a wide range of potential technological applications including that of solid state lighting, power electronics and MEMS devices^7–9^. Specifically, the invention of violet and blue GaN-based lighting emitting diode (LED) technologies has been recognized by the Nobel Prize in Physics in 2014^10^.

The immense attention directed towards understanding and improving the conventional III-nitride alloys has led to significant breakthrough in the past decade. In comparison to GaN, InGaN and AlGaN alloys, the dilute-anion III-nitride-based material systems have received much less attention in the past decades^11,12^. In other words, the development in the dilute-anion III-nitride semiconductor class is still in the early stage and the understanding of these materials is still at its infancy. One of the prime examples in the dilute-anion III-nitride semiconductor class is the GaNAs with minute amount of arsenic (As) ~1–5%, which is called as “dilute-As GaNAs”; it is important to note that, its counterpart dilute-nitride InGaAsN alloy has been extensively investigated, which had resulted in state-of-the-art low threshold laser for telecommunication applications^13–16^.

The dilute-As GaNAs alloys have been experimentally realized via metal-organic chemical vapor deposition (MOCVD) with As-content reaching up to ~7%^17,18^. The GaNAs alloys with the whole As-content range have also been synthesized using molecular beam epitaxy (MBE) techniques^19^. While the experimental work demonstrated the feasibility to synthesize dilute-As GaNAs alloys, the physical properties of the alloy are still relatively unexplored. Recent work on the dilute-As GaNAs alloy shed light on its electronic properties, pointing out the large band gap coverage from ~2.3 eV to ~3.4 eV with As-content as little as 6%^11,20–23^. In addition, the interband Auger recombination process was suppressed within the dilute-As GaNAs alloy, indicating possible reduction of efficiency droop issue known in the InGaN based LEDs^24^. Thus, a novel design based on the InGaN/GaNAs interface quantum well (QW) has been proposed and it has been shown that the spontaneous emission rate for red emission can be enhanced by about 8.5 times compared to the conventional InGaN QW design^25^. These studies indicate a strong potential for implementing the dilute-As GaNAs semiconductor as the active region material in light-emitting devices.

The investigations of the electronic properties of dilute-As GaNAs alloy have provided a necessary foundation and possible ways for future device implementation. However, the other material properties of dilute-As GaNAs alloys, specifically the optical properties, are yet to be understood. Among the optical properties for example, the refractive indices of semiconductors have played a key role in photonics applications, including that of distributed Bragg reflector (DBR), waveguides and photonics crystals^26–28^. Therefore, intensive understanding of the behavior of the optical properties of the dilute-As GaNAs material is of high importance for future photonic device design.

In this work, we present an analysis of the optical properties of dilute-As GaN_1−x_As_x_ alloys by employing First-Principle Density Functional Theory (DFT) calculations along with the use of a scissor operator. The arsenic (As) atoms are introduced as a replacement of nitrogen (N) atoms in the bulk GaN material system, in which the As-content is varied from 0% up to 12.5% As-content. The imaginary and real parts of the dielectric function of dilute-As GaNAs, with comparison to theoretical and experimental values of GaN are specifically presented and discussed. Moreover, the complex refractive index, normal-incidence reflectivity, as well as birefringence, of the wurtzite material system are presented and comparison is drawn to other III-nitride material systems. Thus, we found that the changes in the refractive index of the GaNAs alloys as compared to the GaN system can be significant, considering that the addition of arsenic is minute in the GaN material.

## Computational Method

**Figure 1:**
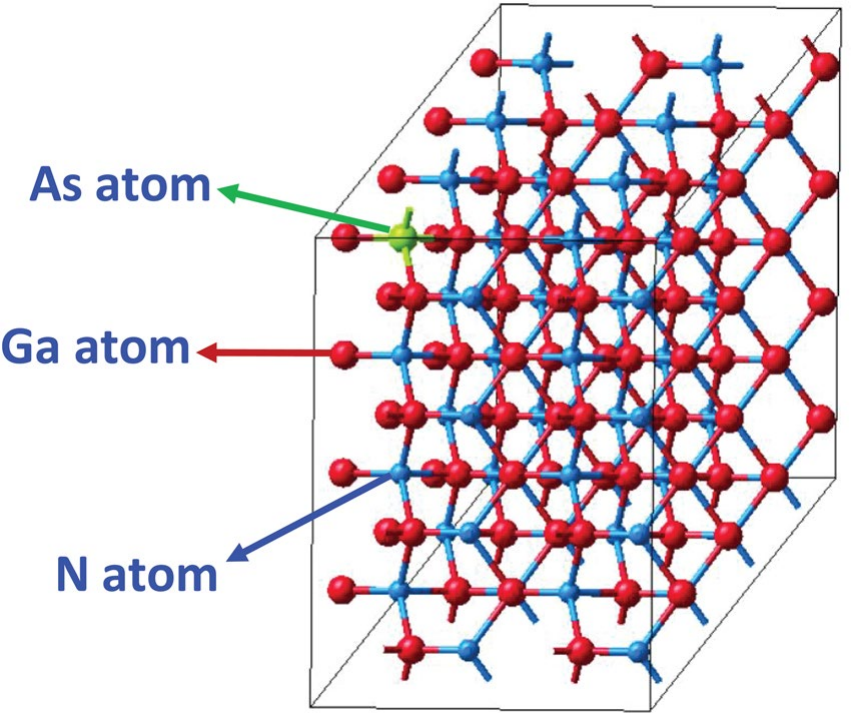
Illustration of a 128-atom dilute-As GaNAs supercell, with 1 arsenic atom replacing a nitrogen atom, corresponding to 1.56% As-content.

The DFT calculations in this study utilized the supercell approach to build the appropriate crystal structures of the dilute-As GaNAs alloys to calculate the optical properties. In Fig. 1, a 128-atom supercell, comprised of 64 gallium (Ga) atoms, 63 nitrogen atoms and 1 arsenic atom is shown. By varying the supercell size and keeping one As-atom in the system at a time, the As-content in the alloys was varied, where the 128-atom supercell corresponds to 1.56% As-content. The projector augmented wave (PAW) method within the Vienna ab initio simulation package (VASP) was used to obtain the optical properties of the GaN_1−x_As_x_ alloys^29,30^. Moreover, the DFT calculations utilized the Generalized Gradient Approximation (GGA) and the Perdew-Burke-Ernzerhof (PBE) exchange-correlation functional for optimizing the structure^31^. Thus, the optical properties were then calculated using the Local Density Approximation (LDA), where a scissor operator has been applied to shift the imaginary part of the dielectric function, similar to previous works^32^. The cut-off energy was set to 400 eV and the external stress applied to the system was set to 0 GPa. Structure optimization was done using a Hellman-Feynman force of 0.02 eV/Å and an energy convergence criterion of 1 × 10^−5^ eV/atom. Our calculations are performed based on the blocked Davidson algorithm while the tetrahedron smearing method was used as the integration scheme for the optical spectra. In addition, the number of bands and k-mesh sizes vary depending on the supercell size. As an example, different k-meshes were generated where the k-mesh for 128-atom supercell had a 3 × 3 × 3 mesh, the 64-atom had a 7 × 7 × 7 mesh and the 16-atom supercell had a 9 × 9 × 9 mesh. Furthermore, we performed calculations including the spin-orbit coupling effect, to determine the effect of arsenic, but the results showed a negligible effect, similar to other wide band-gap III-nitride semiconductors and therefore has been excluded from the calculations in this work.

### Complex Dielectric Function for Dilute-As GaNAs Alloys

The complex dielectric function is defined as *ε*(*E*) = *ε*_1_(*E*) + *iε*_2_(*E*), where *ε*_1_(*E*) and *ε*_2_(*E*) are the real and imaginary parts of the complex dielectric function, respectively. The real and imaginary parts of the dielectric function are related by the Kramers-Kronig transformation in the following way^33^:

1 $${\varepsilon }_{1}(E)=1+\frac{2}{\pi }{\int }_{0}^{\infty }\frac{E^{\prime} {\varepsilon }_{2}(E^{\prime} )}{E{^{\prime} }^{2}-{E}^{2}}dE^{\prime} $$

2 $${\varepsilon }_{2}(E)=\,\frac{-2E}{\pi }{\int }_{0}^{\infty }\frac{{\varepsilon }_{1}(E^{\prime} )}{E{^{\prime} }^{2}-{E}^{2}}dE^{\prime} $$

Therefore, once the imaginary part is obtained, it is relatively straight forward to obtain the real part of the dielectric function and vice versa.

**Figure 2:**
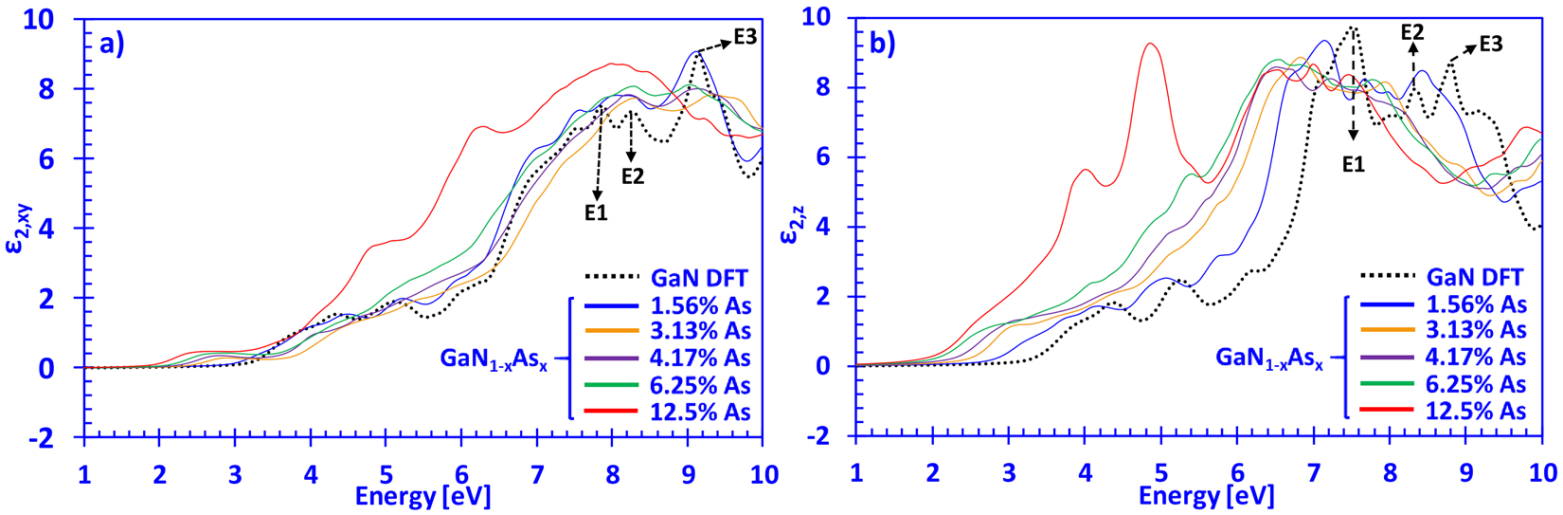
(**a**) Imaginary part of the dielectric in the xy-direction (*ε*_2,*xy*_(*E*)) and (**b**) z-direction (*ε*_2,*xy*_(*E*)) spectra for dilute-As GaNAs alloys, with As-content ranging from 0% up to 12.5% arsenic, respectively.

Figure 2(a) and (b) show the imaginary parts of the complex dielectric function for dilute-As GaNAs alloys with As-content ranging from 0% up to 12.5%, for the xy- and z-directions, respectively. Moreover, the xy-direction is defined as the polarization vector perpendicular (**E ⊥ c)** to the surface of the GaNAs alloy, whereas the z-direction corresponds case parallel (**E** || **c)** to the surface (c-axis) of the GaNAs alloy. Hence, the dilute-As GaNAs alloys exhibit anisotropy, similar to GaN and other ternary III-nitride material systems such as InGaN and AlGaN^34,35^.

From Fig. 2(a), increasing the arsenic content in the dilute-As GaNAs alloys leads to a shift in the onsets of the imaginary parts of the dielectric functions in the xy-direction towards lower photon energies. This phenomenon is analogous to the decrease in the energy band gap, which has been reported previously in the literature^11,36^. In addition, the positions of the E1, E2 and E3 absorption peaks of GaN in Fig. 2(a) can be observed at approximately 7.8 eV, 8.3 eV and 9.1 eV, respectively. The locations, as well as the magnitudes of the peaks compare well to those previously reported by Benedict and Shirley (LDA), whose peaks were around 7.5 eV, 7.9 eV, and 9 eV^37^. Similarly, Laskowski and co-workers (GGA) obtained peaks located around 7.5 eV, 7.9 eV and 9 eV for the E1, E2 and E3 peaks, respectively^38^. Moreover, increasing the arsenic content shifts the main absorption peaks of *ε*_2,*xy*_(*E*) of the dilute-As GaNAs alloys towards the lower energy spectrum, with the 12.5% As-containing alloy showing the most prominent change in the overall shape of the *ε*_2,*xy*_(*E*) spectra.

The *ε*_2,*z*_(*E*) spectra for GaN in Fig. 2(b) shows the E1, E2 and E3 absorption peaks located at around 6.9 eV, 8.1 eV and 8.9 eV, respectively. These values are in good agreement with the results of Laskowski and co-workers, who obtained peaks around 7.2 eV, 8 eV and a broad peak around 9 eV, for the E1, E2 and E3 peaks, respectively. In addition, Benedict and Shirley have reported the respective peaks located around 7 eV, 8 eV and 9 eV, for the case of parallel polarized imaginary part of the dielectric function, which agree well with our results. The good agreement on the imaginary part of the dielectric function for GaN with previous works provides a strong confidence on the method we used in this work to obtain the optical properties of the dilute-As GaNAs alloys. Moreover, increasing arsenic content in the dilute-As GaNAs alloys has more prominent changes in the magnitude of *ε*_2,*z*_(*E*) spectra (Fig. 2(b)) compared to the perpendicular polarized case (Fig. 2(a)), up to the 6 eV region. Thus, the shift in the main absorption peaks (E1-E3) of *ε*_2,*z*_(*E*) spectra for GaNAs alloys containing 1.56% arsenic up to 12.5% are incremental with respect to DFT-calculated GaN.

**Figure 3:**
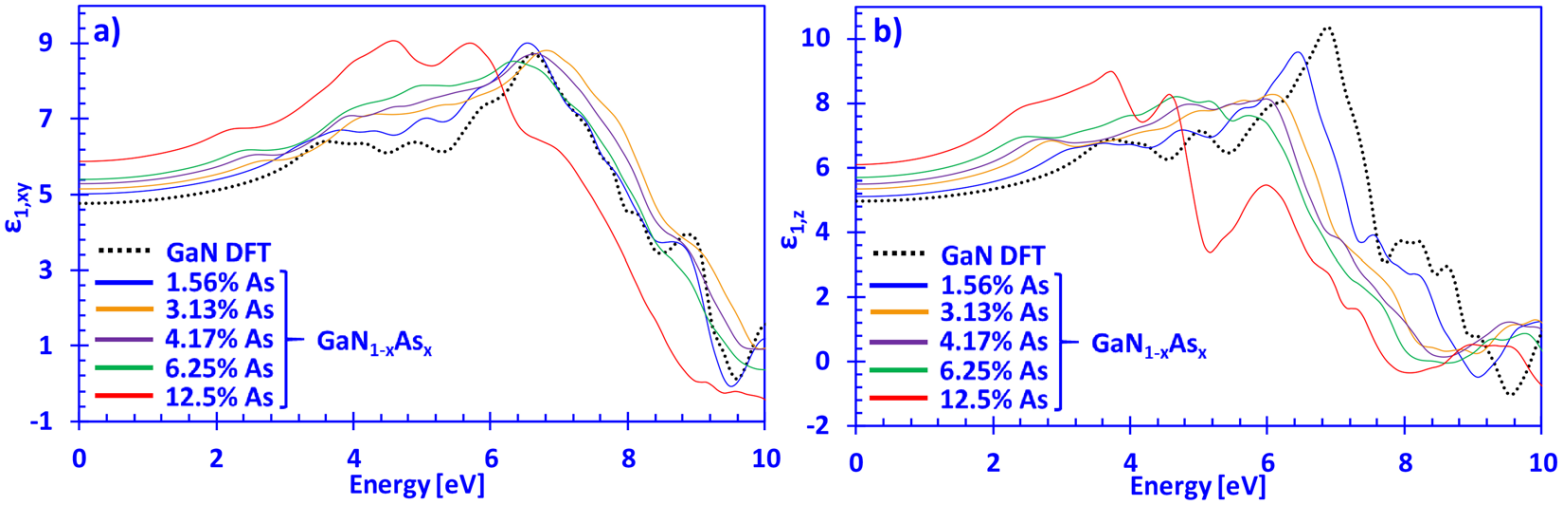
(**a**) Real part of the dielectric function in the xy-direciton (*ε*_1,*xy*_(*E*)) and (**b**) z-direction (*ε*_1,*z*_(*E*)) spectra for dilute-As GaNAs alloys, with As-content ranging from 0% up to 12.5% arsenic, respectively.

Figure 3(a) and (b) show the calculated real part of the dielectric function of dilute-As GaNAs alloys, with As-content ranging from 0% up to 12.5%, for the xy- and z-directions, respectively. Note that the real part of the complex dielectric function was obtained by using equation (1), as mentioned above. The values of *ε*_1,*xy*_(*E*) for GaN are in good agreement with theoretical works from Persson and co-workers, as well as the experimental and theoretical report from Benedict and co-workers^39,40^. Figure 3(a) indicates that for the *ε*_1,*xy*_(*E*) spectra, increasing the arsenic content leads to significant enhancements in the magnitude of the real part of the dielectric function, as compared to GaN. In addition, the high-frequency dielectric constant (from Fig. 3(a)) for the xy-direction for GaN was deduced to be *ε*_*xy*_(∞)~4.8, which compares well to previously reported theoretical values from Lambrecht and co-workers (~4.79), but is slightly lower compared to Takeuchi and co-workers (~5.27) and de Carvalho and co-workers (~5.11)^41–43^. Thus, Fig. 3(a) indicates that increasing the As-content in the dilute-As GaNAs alloys leads to higher values of the high-frequency dielectric constant (*ε*_*xy*_(∞)), with the 12.5% As-content alloy having a value of *ε*_*xy*_(∞) ~5.85. Additionally, the static dielectric constant ε_*s,xy*_ for DFT-calculated GaN is ~8.7, which is in good agreement with experimental results found by Strite and Morkoç (~8.9)^44^. On the other hand, increasing the arsenic content leads to slightly higher values in the static dielectric constant, with the 12.5% As-containing alloy having ε_*s,xy*_ ~9. Although these results have not yet been experimentally confirmed, they can be useful in simulation modelling for future photonic device applications.

On the other hand, Fig. 3(b) indicates that the **E** ‖ **c** polarized real part of the dielectric function of our DFT-calculated results is in good agreement with those of Benedict and co-workers^40^. The DFT-calculated GaN high-frequency dielectric constant for the z-direction resulted in a value of *ε*_*z*_(∞) ~4.95 - which is slightly higher than that reported by Christensen *et al*. (~4.62) and slightly lower compared to de Carvalho *et al*. (~5.3)^43,45^. The discrepancy can be ascribed to the difference in the method for calculating the *ε*_2_(*E*) spectra, as Christensen *et al*. did not include any LDA correction terms. Thus, compared to Fig. 3(a), Fig. 3(b) shows that the magnitudes of the dielectric functions in the z-direction are slightly higher for all the presented cases as compared to the xy-direction. This trend, which is directly related to the anisotropic behavior of wurtzite material systems, has been observed experimentally in various works including those of Shokhovets, Pezzagna, Rigler with co-workers, and theoretically by Christensen *et al*.^45–48^. Thus, Fig. 3 shows that the high-frequency dielectric function for all *ε*_*z*_(∞) values is higher compared to *ε*_*xy*_(∞) for all dilute-As GaNAs alloy compositions studied, and that the increase is a linear relationship of arsenic content. This indicates a possibility allowing for precise control of the optical properties of the GaN-based alloy by simply tuning the arsenic content.

### Complex Refractive Index, Reflectivity and Birefringence of Dilute-As GaNAs Alloys

By using the information obtained from the complex dielectric function, optical constants like the complex refractive index *n* ^*^(*E*) = *n*(*E*) + *ik*(*E*) can be obtained with the following relations^33^:

3 $$n(E)=\sqrt{\frac{\sqrt{{\varepsilon }_{1}{(E)}^{2}+{\varepsilon }_{2}{(E)}^{2}}+{\varepsilon }_{1}{(E)}^{2}}{2}}$$

4 $$k(E)=\sqrt{\frac{\sqrt{{\varepsilon }_{1}{(E)}^{2}+{\varepsilon }_{2}{(E)}^{2}}-{\varepsilon }_{1}{(E)}^{2}}{2}}$$

**Figure 4:**
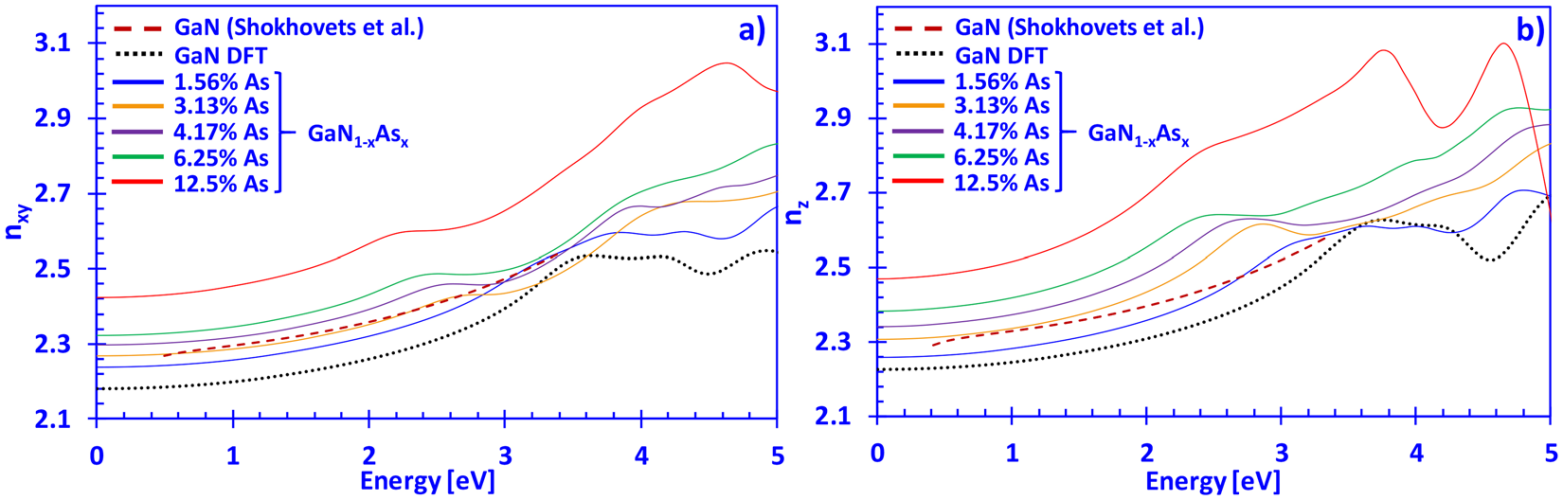
(**a**) Refractive index in the xy-direction (*n*_*xy*_(*E*)) and (**b**) z-direction (*n*_*z*_(*E*)) spectra for dilute-As GaNAs alloys (solid lines), with As-content ranging from 0% up to 12.5% arsenic, and experimentally fitted data (dashed lines), respectively.

Figure 4(a) and (b) show the refractive index for the xy- (*n*_*xy*_(*E*)) and z-directions (*n*_2_(*E*)) for DFT-calculated dilute-As GaNAs alloys, with As-content varying from 0% up to 12.5%, along with experimentally fitted data for GaN, respectively. Figure 4(a) indicates the refractive index in the xy-direction increasing linearly as the arsenic content in the dilute-As GaNAs alloys is increased for photon energies up to ~3.5 eV. Moreover, the E0 peak (band edge) of DFT-calculated GaN is approximately located around ~3.4 eV, which agrees well with data reported by Takeuchi and Djurišić with co-workers^41,49^. The respective E0 peaks for dilute-As GaNAs alloys shift to lower photon energies as more arsenic is introduced into the system, with the 12.5% As-containing alloy having the E0 peak around ~2.2 eV. Furthermore, Fig. 4(a) shows that the DFT-calculated refractive index of GaN (n_xy_) is about ~5% on average lower (below the band gap) compared to experimentally measured values of Shokhovets and co-workers^46^. It should be noted that our calculations were performed at 0 K and that effects like strain and surface roughness were not taken into account, albeit previously identified to affect the magnitude of the dielectric function^42,50^. In addition, Rigler and co-workers noted that Ga-polar and N-polar thin films have different refractive indices, which they ascribed it to an effect due to different impurity contents^48^. The effects of temperature on the refractive index of dilute-As GaNAs alloys will be essential for future photonic device design, but this study is not within the scope of this work and will require further investigations^51^.

Figure 4(b) indicates that the experimental values of *n*_*z*_(*E*) from Shokhovets and co-workers for GaN are about ~5% higher on average compared to the values of DFT-calculated GaN, in the energy range from ~0.5 eV up to ~3.3 eV. Similar to the xy-direction, the E0 peaks of the refractive indices in the z-direction of dilute-As GaNAs alloys shifted to the left, as more arsenic is introduced into the GaN-based system, with the 12.5% As-containing alloy showing a peak around ~2.3 eV. Thus, by inserting a minute amount of arsenic into the GaN material system, it is possible to alter the material system in such a way to achieve controlled large refractive index differences compared to GaN. On the other hand, in a material system like InGaN, it has been reported that the refractive index is strongly dependent on numerous factors including phase-separation, the piezoelectric-field induced Stark-effect and variations due to quantum-confinement, making it difficult to predict the refractive index based solely on the indium content^34,52,53^. Furthermore, AlGaN has a similar behavior to dilute-As GaNAs alloys, where the refractive index is a linearly decreasing function for increasing Al-content^41,48^. It should be noted that Fig. 4(a) and (b) do not indicate the alloy compositions that may be absorbing light, for example, the GaN_0.875_As_0.125_ alloy will be absorbing the 450 nm light, since the bandgap is around ~2.23 eV.

**Figure 5:**
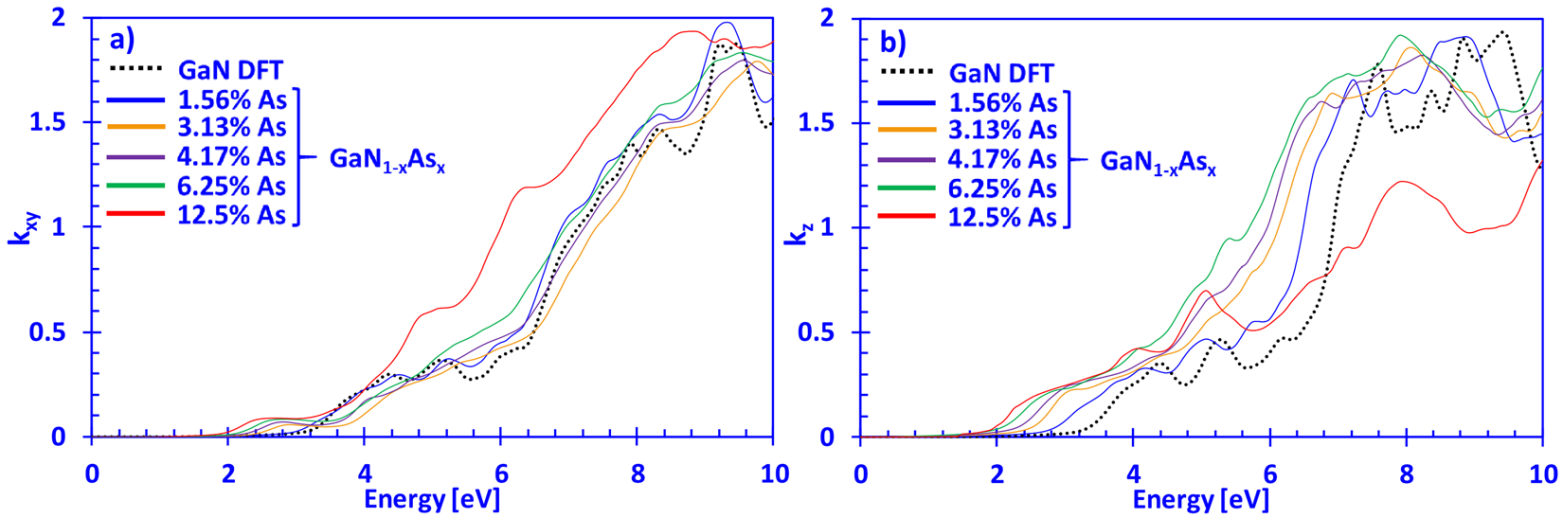
(**a**) *k*_*xy*_(*E*) and (**b**) *k*_*z*_(*E*) spectra for dilute-As GaNAs alloys, with As-content ranging from 0% up to 12.5% arsenic.

Figure 5(a) and (b) present the imaginary part of the complex refractive index data in the xy- and z-directions for dilute-As GaNAs alloys with As-content varying from 0% up to 12.5%, respectively. For the *k*_*xy*_(*E*) spectra in Fig. 5(a), the absorption onset is noticeable around ~3.5 eV, which is consistent with the reported values of the energy band gap for GaN. Moreover, the overall shape and magnitude of our DFT-calculated and the GaN data reported by Lambrecht *et al*. are in good agreement for energies up to 10 eV^42^. Furthermore, increasing the As-content in dilute-As GaNAs alloys shifts the *k*_*xy*_(*E*) spectra towards the lower photon energy region, consistent with the observed energy gap reduction from previous works^11,36^. Thus, in Fig. 5(a), the overall magnitude of *k*_*xy*_(*E*) increases as more arsenic is introduced into the dilute-As GaNAs system, which is not the case for the data presented in Fig. 5(b). Namely, the 12.5% As-containing GaNAs alloy has a noticeably lower *k*_*z*_(*E*) value in the range from 7 eV to 10 eV compared to all other systems considered. In addition, the overall shape, of *k*_*xy*_(*E*) and *k*_*z*_(*E*) of DFT-calculated GaN coincides well with previous theoretical works of Lambrecht and co-workers, as well as Djurišić and Takeuchi with co-workers, although the magnitude is slightly lower in our case as compared to the latter two^41,42,49^.

By using the results on the complex refractive index, it is possible to extract the normal-incidence reflectivity for the dilute-As GaNAs alloys by employing the following relation:

5 $$R(E)=\frac{{[n(E)-1]}^{2}+k{(E)}^{2}}{{[n(E)+1]}^{2}+k{(E)}^{2}}$$

**Figure 6:**
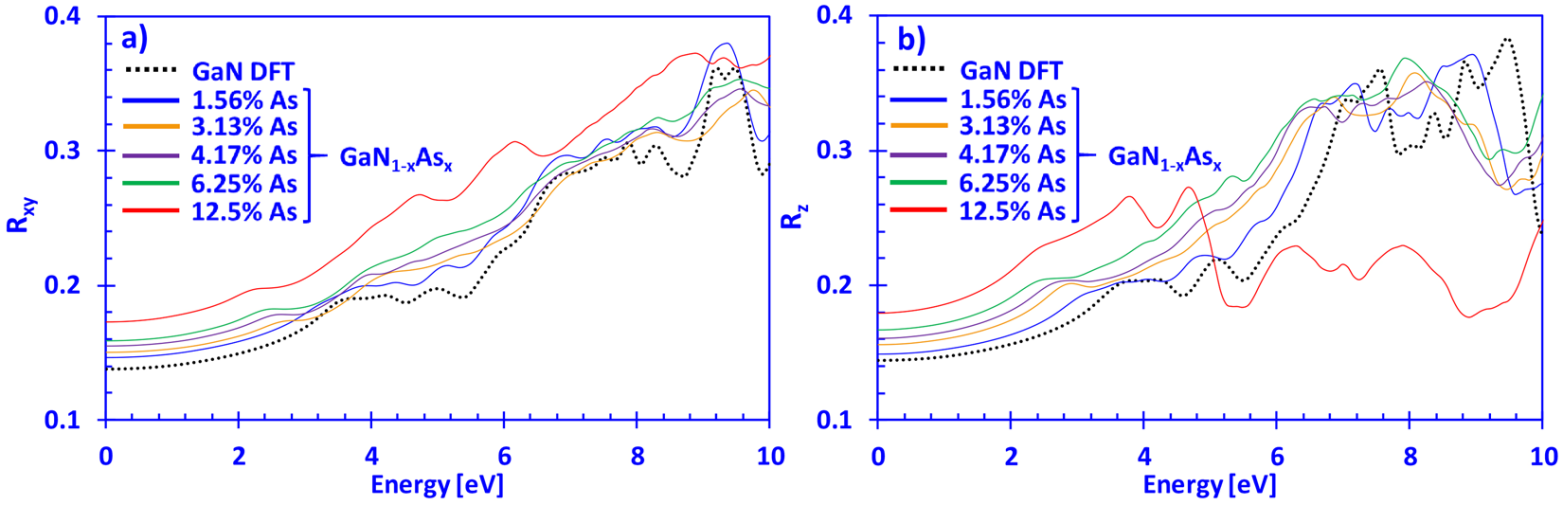
(**a**) *R*_*xy*_(*E*) and (**b**) *R*_*z*_(*E*) spectra for dilute-As GaNAs alloys, with As-content ranging from 0% up to 12.5% arsenic.

Figure 6(a) and (b) present the normal-incidence reflectivity for dilute-As GaNAs alloys with As-contents ranging from 0% up to 12.5%, for the xy- and z-directions, respectively. From Fig. 6(a), the reflectivity for DFT-calculated GaN in the xy-direction (*R*_*xy*_(*E*)) falls in the range of ~0.13–0.18 for energy ranging from 0 eV to 3 eV, which is in good agreement with the values reported by Kawashima and co-workers^54^. Similarly, Fig. 6(b) also indicates that the normal-incidence reflectivity for DFT-calculated GaN in the z-direction (*R*_*z*_(*E*)) lies in the range of 0.14–0.18. On the other hand, the 12.5% As-containing GaNAs alloy has the highest *R*_*xy*_(*E*) and is varying between ~0.17–0.2, within the visible regime. Thus, when the arsenic content in the dilute-As GaNAs alloys is increased, a linear enhancement in the normal-incidence reflectivity for the xy-, as well as z-directions is observed, as shown in Fig. 6(a) and (b), for energies up to ~3 eV.

**Figure 7:**
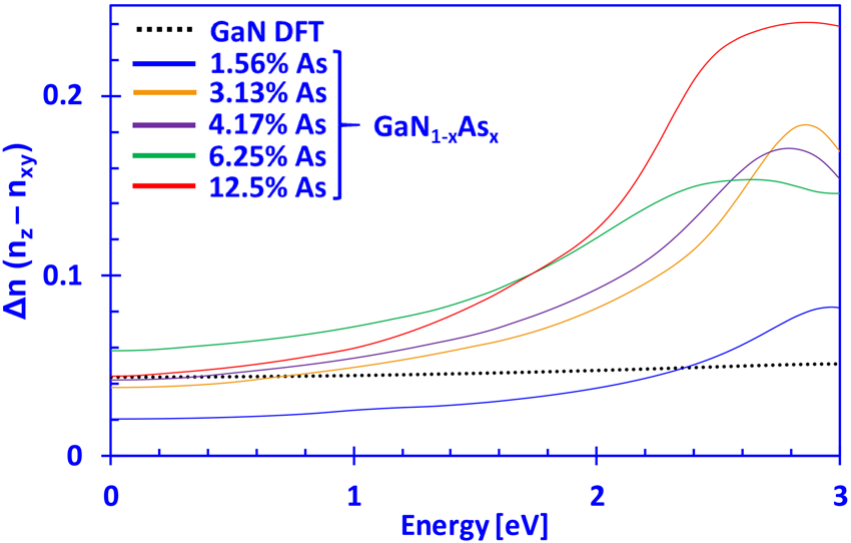
Birefringence (Δn) of dilute-As GaN_1−x_As_x_ alloys with As-content ranging from 0% up to 12.5%.

The birefringence of the dilute-As GaNAs alloy can be extracted out by comparing its real part of the complex refractive indices from both directions (xy and z). The birefringence is defined as Δn = n_z_ – n_xy_, where n_z_ is the refractive index in the z-direction and n_xy_ is the refractive index in the xy-direction. Figure 7 presents the birefringence (Δn) as a function photon energy of dilute-As GaN_1−x_As_x_ alloys, where the arsenic content varies from 0% up to 12.5%. The birefringence of DFT-calculated GaN aligns well with previously reported experimental values where a value of Δn ~0.0425 by Pezzagna and co-workers was obtained, whereas Sanford and co-workers reported Δn ~0.038^47,55^. Hui and co-workers reported Δn ~0.04 and Shokhovets and co-workers have reported a varying birefringence, that increases with increasing photon energy and ranges between 0.037 and 0.056^46,56^. In this work, we obtained a birefringence for GaN that increases slowly from 0.044 to 0.051 for energies approaching the band edge. In addition, Rigler and co-workers have reported that birefringence may vary depending whether the GaN thin films are Ga-polar or N-polar, with the Ga-polar exhibiting slightly higher refractive indices – something that has not been taken into account in this work^48^. On the other hand, it is important to notice that the birefringence of the dilute-As GaNAs alloys can be as low as ~0.02 when the As-content is relatively minute (1~2%). However, while approaching the bandgap value (~3.3 eV) for GaNAs with 1.56% As-content, its birefringence increases and exceeded that of GaN. This feature may prove interesting, as it may indicate a potential pathway to reduce the birefringence in the nitride-based system by simply inserting low amounts of arsenic into the GaN system. Additional work is necessary to address this reduction and determine the influence of temperature on the birefringence, as well as the refractive index. In general, our results indicate an increasing trend in Δn for higher As-content in the dilute-As GaNAs alloy.

## Conclusion

In summary, First-Principle DFT calculations were carried out to evaluate the optical properties of dilute-As GaNAs semiconductor alloys with As-content ranging from 0% to 12.5%. By using the knowledge on the complex dielectric function, optical constants including the complex refractive index and normal-incidence reflectivity were evaluated and analyzed. Our findings showed that dilute-As GaNAs alloys are optically anisotropic, and the analysis on the birefringence of the alloys was compared to other conventional III-nitride material systems. In addition, our analysis indicated large index difference (up to ~0.35) between dilute-As GaNAs alloy and GaN alloy across a large range of photon energies. Specifically, the refractive index of dilute-As GaNAs had a linear relationship with respect to the arsenic content in the alloys, indicating potential increment at even higher arsenic contents. The large refractive index difference tuning capability between GaN and dilute-As GaNAs alloys could prove beneficial for the design of waveguides and distributed Bragg reflectors, requiring the use of large refractive index differences. Our work points out the promising optical properties of dilute-As GaNAs semiconductors and pose them as strong candidates for materials for photonic device applications.

## References

[1] Morkoç, H. Nitride Semiconductor Devices: Fundamentals and Applications, (Wiley-VCH Weinheim, 2013).

[2] Nakamura, S. & Fasol, G., (Eds.), *The Blue Laser Diode*, (Springer-Verlag, Berlin 1997).

[3] Crawford, M. H. “LEDs for solid-state lighting: performance challenges and recent advances”, *IEEE J. Sel. Top*. Quantum Electron., vol. **15**, pp. 1028–1040, August (2009).

[4] Tsao JY (2004). “Toward Smart and Ultra-efficient Solid-State Lighting”. Adv. Opt. Mat..

[5] Harima H (2002). “Properties of GaN and related compounds studied by means of Raman scattering”. J. Phys.: Condens. Matter.

[6] Zeng G, Tan CK, Tansu N, Krick BA (2016). “Ultralow wear of gallium nitride”. Appl. Phys. Lett..

[7] Mishra UK, Shen L, Kazior TE, Wu YF (2008). “GaN-Based RF Power Devices and Amplifiers”. Proc. IEEE.

[8] Wiesmann, C., Bergenek, K., Linder, N. & Schwarz, U. T. “Photonic crystal LEDs – designing light extraction”, Laser & Photon. *Rev*., vol. **3**, no. 3, pp. 262–286, (2009).

[9] Cimalla, V., Pezoldt, J. & Ambacher, O. “Group III nitride and SiC based MEMS and NEMS: materials properties, technology and applications”, *J. Phys. D: Appl. Phys*., vol. **40**, 6386, October (2007).

[10] http://www.nobelprize.org/nobel_prizes/physics/laureates/2014/

[11] Tan, C. K. *et al*. “First-Principle electronic properties of dilute-As GaNAs alloy for visible light emitters”, *J. Disp. Tech*., vol. **9**, no. 4, pp 272–279, April (2013).

[12] Tan, C. K., Borovac, D., Sun, W. & Tansu, N. “First-Principle Electronic Properties of Dilute-P GaN_1−x_P_x_ Alloy for Visible Light Emitters”, *Sci. Rep*., vol. **6**, 24412, April (2016). 10.1038/srep24412 PMC4830964 27076266

[13] Choi, W. J., Dapkus, P. D. & Jewell, J. J. “1.2-μm GaAsP/InGaAs strain compensated single-quantum-well diode laser on GaAs using metal organic chemical vapor deposition”, vol. **11**, no. 12, pp. 1572–1574, December (1999).

[14] Ekins-Daukes, N. J. *et al*. “Strain-balanced GaAsP/InGaAs quantum well solar cells”, *Appl. Phys. Lett*., vol. **75**, no. 26, pp. 4195–4197, December (1999).

[15] Tansu, N. & Mawst, L. J. “High-performance, strain compensated InGaAs-GaAsP-GaAs (λ = 1.17 μm) quantum well diode lasers”, IEEE Photon. *Technol. Lett*., vol. 13, no. 3, pp. 179–181, March (2001).

[16] Hou, H. Q., Choquette, K. D., Geib, K. M. & Hammons, B. E. “High-performance 1.06-μm selectively oxidized vertical-cavity surface-emitting lasers with InGaAs-GaAsP strain-compensated quantum wells”, *IEEE Photo. Technol. Lett*., vol. 9, no. 8, pp. 1057–1059, August (1997).

[17] Li, X., Kim, S., Reuter, E. E., Bishop, S. G. & Coleman, J. J. “The incorporation of arsenic in GaN by metalorganic chemical vapor deposition”, *Appl. Phys. Lett*., vol. **72**, pp. 1990–1992, (1998).

[18] Kimura, A., Paulson, C. A., Tang, H. F. & Kuech, T. F. “Epitaxial GaN_1-y_As_y_ layers with high As content grown by metalorganic vapor phase epitaxy and their band gap energy”, *Appl. Phys. Lett*., vol. **84**, no. 9, pp. 1489–1491, March (2004).

[19] Novikov, S. V. *et al*. “Growth by molecular beam epitaxy of amorphous and crystalline GaNAs alloys with band gaps from 3.4 to 0.8 eV for solar energy conversion devices”, Journal of Crys. Growth, vol. **323**, 1, pp. 60-63, May (2011).

[20] Tan, C. K. & Tansu, N. “First-Principle natural band alignment of GaN/dilute-As GaNAs alloy”, *AIP Advances*, vol. **5**, no.1, p. 071129, January (2015).

[21] Tan, C. K. & Tansu, N. “Nanostructured Lasers: Electrons and Holes Get Closer”, *Nature Nanotechnology*, vol. **10**, pp. 107–109 (2015). 10.1038/nnano.2014.333 25599192

[22] Arif, R. A., Zhao, H. & Tansu, N. “Type-II InGaN-GaNAs quantum wells active regions for lasers applications”, *Appl. Phys. Lett*., vol. **92**, p. 011104, January (2008).

[23] Zhao, H. P., Arif, R. & Tansu, N. “Self-consistent gain analysis of type-II ‘W’ InGaN-GaNAs quantum well lasers”, *J. Appl. Phys*., vol. **104**, 043104, August (2008).

[24] Tan, C. K. & Tansu, N. “Auger recombination rates in dilute-As GaNAs semiconductor”, *AIP Advances*, vol. **5**, no. 5, p. 057135, May (2015).

[25] Tan, C. K., Borovac, D., Sun, W. & Tansu, N. “InGaN/Dilute-As GaNAs Interface Quantum Well for Red Emitters”, *Sci. Rep*., vol. **6**, 19271, January (2016). 10.1038/srep19271 PMC4725840 26758552

[26] Ng, H. M., Moustakas, T. D. & Chu, S. N. G. “High reflectivity and broad bandwidth AlN/GaN distributed Bragg reflectors grown my molecular beam epitaxy”, *Appl. Phys. Lett*., vol. **76**, 2818, May (2000).

[27] Gromovyi M, Semond F, Duboz JY, Feuillet G, De Micheli MP (2014). “Low loss GaN waveguides for visible light on Si substrates”. J. Europ. Opt. Soc. Rap. Public..

[28] Oder, T. N., Shakya, J., Lin, J. Y. & Jiang, H. X. “III-nitride photonic crystals”, *Appl. Phys. Lett*., vol. **83**, no. 6, August (2003).

[29] MedeA-VASP, Material Designs Inc.

[30] Kresse, G. & Furthmuller, J. “Efficient iterative schemes for ab initio total-energy calculations using a plane-wave basis set”, *Phys. Rev. B*., vol. **54**, no. 16, pp. 11169–11186, October (1996). 10.1103/physrevb.54.11169 9984901

[31] Perdew, J. P., Burke, K. & Ernzerhof, M. “Generalized Gradient Approximation Made Simple”, *Phys. Rev. Lett*., vol. **77**, no. 18, October (1996). 10.1103/PhysRevLett.77.3865 10062328

[32] Del Sole, R. & Girlanda, R. “Optical properties of semiconductors within the independent-quasiparticle approximation”, *Phys. Rev. B*, vol. **48**, no. 16, October (1993). 10.1103/physrevb.48.11789 10007516

[33] Adachi, S. *Properties of Semiconductor Alloys: Group-IV, III-V nad II-VI Semiconductors* (Wiley, Chichester, 2009).

[34] Sanford, N. A. *et al*. “Refractive index and birefringence of In_x_Ga_1–x_N films grown by MOCVD”, *Phys. Stat. Solidi (C)* **2**, no. 7 (2005).

[35] Özgür Ü, Webb-Wood G, Everitt HO, Yun F, Morkoç H (2001). “Systematic measurement of AlxGa1ÀxN refractive indices”. Appl. Phys. Lett..

[36] Wu, J. *et al*. “Valence band hybridization in N-rich GaN_1−x_As_x_ alloys”, *Phys. Rev. B*, vol. **70**, no. 11, p.115214, September (2004).

[37] Benedict, L. X. & Shirley, E. L. “*Ab initio* calculation of ε_2_ (ω) including the electron-hole interaction: Application to GaN and CaF_2_”, *Phys. Rev. B*, vol. **59**, no. 8, February (1999).

[38] Laskowski, R., Christensen, N. E., Santi, G. & Ambrosch-Draxl, C. “*Ab initio* calculations of excitons in GaN”, *Phys. Rev. B*, vol. **72**, 0352404, July (2005).

[39] Persson, C., Ahuja, R., Ferreira da Silva, A. & Johansson, B. “First-principle calculations of optical properties of wurtzite AlN and GaN”, J. of Crystal Growth, vol. **231**, pp. 407–414, (2001).

[40] Benedict, L. X. *et al*. “Dielectric function of wurtzite GaN and AlN thin films”, *Solid State Communications*, vol. **112**, pp. 129–133, (1999).

[41] Takeuchi, K., Adachi, S. & Ohtsuka, K. “Optical properties of Al_x_Ga_1−x_N alloy”, *J. Apply. Phys*., vol. **107**, 023306, January (2010).

[42] Lambrecht, W. R. L., Seagall, B., Rife, J., Hunter, W. R. & Wickenden, D. K. “UV reflectivity of GaN: Theory and experiment”, *Phys. Rev. B*, vol. **51**, no. 19, May (1995). 10.1103/physrevb.51.13516 9978155

[43] de Carvalho, L. C., Schleife, A., Furthmüller, J. & Bechstedt, F. “*Ab initio* calculation of optical properties with excitonic effects in wurtzite In_x_Ga_1−x_N and In_x_Al_1−x_N alloys”, *Phys. Rev. B*, vol. **87**, 195211, May (2013).

[44] Strite, S. & Morkoç, H. “GaN, AlN, and InN: A review”, *J. Vac. Sci. Technol. B*, vol. **10**, 1237, August (1992).

[45] Christensen, N. E. & Gorczyca, I. “Optical and structural properties of III-V nitrides under pressure”, *Phys. Rev. B*, vol. **50**, no. 7, August (1994). 10.1103/physrevb.50.4397 9976740

[46] Shokhovets S, Himmerlich M, Kirste L, Leach JH, Krischok S (2015). “Birefringence and refractive indices of wurtzite GaN in the transparency range”. Appl. Phys. Lett..

[47] Pezzagna, S., Brault, J., Leroux, M., Massies, J. & de Micheli, M. “Refractive indices and elasto-optic coefficients of GaN studied by optical waveguiding”, *Journal of Appl. Phys*., vol. **103**, 123112, June (2008).

[48] Rigler M (2013). “Refractive index of III-metal polar and N-polar AlGaN waveguides grown by metal organic chemical vapor deposition”. Appl. Phys. Lett..

[49] Djurišić, A. B., & Li, E. H. “Modeling the optical constants of hexagonal GaN, InN, and AlN”, *J. Appl. Phys*., vol. **85**, 2848, March (1999).

[50] Logothetidis, S., Petalas, J., Cardona, M., & Moustakas, T. D. “Optical properties and temperature dependence of the interband transitions of cubic and hexagonal GaN”, *Phys. Rev. B*, vol. **50**, 18 017 (1994). 10.1103/physrevb.50.18017 9976231

[51] Tisch, U., Meyler, B., Katz, O., Finkman, E. & Salzman, J. “Dependence of the refractive index of Al_x_Ga_1−x_N on temperature and composition at elevated temperatures”, *Journal of Appl. Phys*., vol. **89**, no. 5, March (2001).

[52] McCluskey, M. D. *et al*. “Phase separation in InGaN/GaN multiple quantum wells”, *Appl. Phys. Lett*., vol. **72**, 1730, April (1998).

[53] Doppalapudi, D., Basu, S. N., Ludwig, K. F. Jr. & Moustakas, T. D., “Phase separation and ordering in InGaN alloys grown by molecular beam epitaxy”, *J. Appl. Phys*., vol. **84**, 1389, August (1998).

[54] Kawashima, T., Yoshikawa, H., Adachi, S., Fuke, S. & Ohtsuka, K. “Optical properties of hexagonal GaN”, *Journal of Appl. Phys*., vol. **82**, no. 7, October (1997).

[55] Sanford NA (2003). “Refractive index study of AlxGa1ÀxN films grown on sapphire substrates”. Journal of Appl. Phys..

[56] Hui R (2003). “Birefringence of GaN/AlGaN optical waveguides”. Appl. Phys. Lett..

